# Low-Cost Multispectral System Design for Pigment Analysis in Works of Art

**DOI:** 10.3390/s21155138

**Published:** 2021-07-29

**Authors:** Tania Kleynhans, David W. Messinger, Roger L. Easton, John K. Delaney

**Affiliations:** 1 Chester F. Carlson Center for Imaging Science, Rochester Institute of Technology, Rochester, NY 14623, USA; dwmpci@rit.edu (D.W.M.); easton@cis.rit.edu (R.L.E.J.); 2 National Gallery of Art, Washington, DC 20565, USA; J-Delaney@nga.gov

**Keywords:** hyperspectral, pigment identification, system trade study, band selection study, multispectral

## Abstract

To better understand and preserve works of art, knowledge is needed about the pigments used to create the artwork. Various noninvasive techniques have been used previously to create pigment maps, such as combining X-ray fluorescence and hyperspectral imaging data. Unfortunately, most museums have limited funding for the expense of specialized research equipment, such as hyperspectral reflectance imaging systems. However, many museums have hand-held point X-ray fluorescence systems attached to motorized easels for scanning artwork. To assist museums in acquiring data that can produce similar results to that of HSI systems, while minimizing equipment costs, this study designed and modeled a prototype system to demonstrate the expected performance of a low-cost multispectral system that can be attached to existing motorized easels. We show that multispectral systems with a well-chosen set of spectral bands can often produce classification maps with value on par with hyperspectral systems. This study analyzed the potential for capturing data with a point scanning system through predefined filters. By applying the system and noise modeling parameters to HSI data captured from a 14th-Century illumination, the study reveals that the proposed multispectral imaging system is a viable option for this need.

## 1. Introduction

To make informed decisions about conservation methods for specific works of art, conservation scientists require knowledge about the artwork that includes (but is not limited to) which pigments and binders were used to create the painting, the substrate, and information about any varnish or previous in-painting. Scholars also use this information to learn more about the artist and working methods of the time. To identify the pigments used in an artwork, various noninvasive imaging methods and algorithms have been developed in recent years. The combination of using X-ray fluorescence (XRF) and reflectance hyperspectral imaging (HSI) data has proven very successful in this domain [1,2]. Unfortunately, very few museums can afford high-end HSI systems, and thus, this research has mostly been limited to museums with large conservation centers and university research laboratories. Traveling with valuable artwork to image at these sites is not ideal nor sustainable for acquiring pigment information across an entire collection.

A somewhat less expensive method to collect spectral data is multispectral imaging (MSI). The difference between HSI and MSI lies in the number of spectral channels that is collected (tens versus hundreds). The spectral signature of a material may be more easily identified in HSI, because the spectral resolution is capable of distinguishing finer spectral features that might be missed in a wider-band multispectral image. Due to this difference, MSI data have not often been used to identify pigments in works of art. Studies involving MSI for tentative pigment identification can often identify pure pigments only [3], are used for reconstructing accurate color images and for image classification [4,5], or used on a limited set of paint samples [6]. Note that image classification algorithms identify clusters of similar spectra, but do not label the classes. Assessment by experts and additional imaging are often needed to identify the spectra of the cluster as a pigment or pigment mixture (layered paint structure). MSI and point-XRF measurements have been used successfully on a limited palette at archaeological sites [7]. All systems used in studies with MSI systems either used broadband illumination and spectral filters in front of the lens or illumination with specific LEDs to acquire the required bands of spectral data. However, due to the limited spectral resolution of MSI, which complicates the identification of pigments, research in this space is sparse [3,8].

Often, elemental data of pigments are captured with point-XRF measurement instruments, where small numbers of selected spots on a painting are studied. This obviously is not ideal, as the method necessarily makes assumptions about pigments in areas not imaged. However, many museums recently have acquired motorized scanning easels, with point-XRF instruments mounted to the scanner for acquiring more complete sets of XRF data [9]. For a more comprehensive analysis, HSI data are needed to complement the macroscale XRF data. Unfortunately, HSI systems are still very expensive, and therefore, access typically is currently limited to larger conservation laboratories or research universities. As mentioned, MSI systems are not often used for pigment identification due to insufficient spectral resolution. However, these limitations may be addressed by choosing specific spectral bands for the system that have been previously identified as useful. The selection is made from prior knowledge of the spectral signatures of pigments that may be encountered in the study.

To this end, a multispectral point-imaging system was designed that can be attached to an existing motorized XRF scanning easel, reducing costs and acquisition time. Note that XRF data acquisition with scanning systems often takes hours, if not days [2]. The spectral bands for the MSI system were chosen after a comprehensive band selection study. The proposed system model was validated using HSI reflectance data collected in a previous study [10]. The painting, a 14th-Century illumination, was studied in detail with various imaging modalities, and the results here were compared to the HSI “ground truth” data.

## 2. Materials and Methods

**Figure 1:**
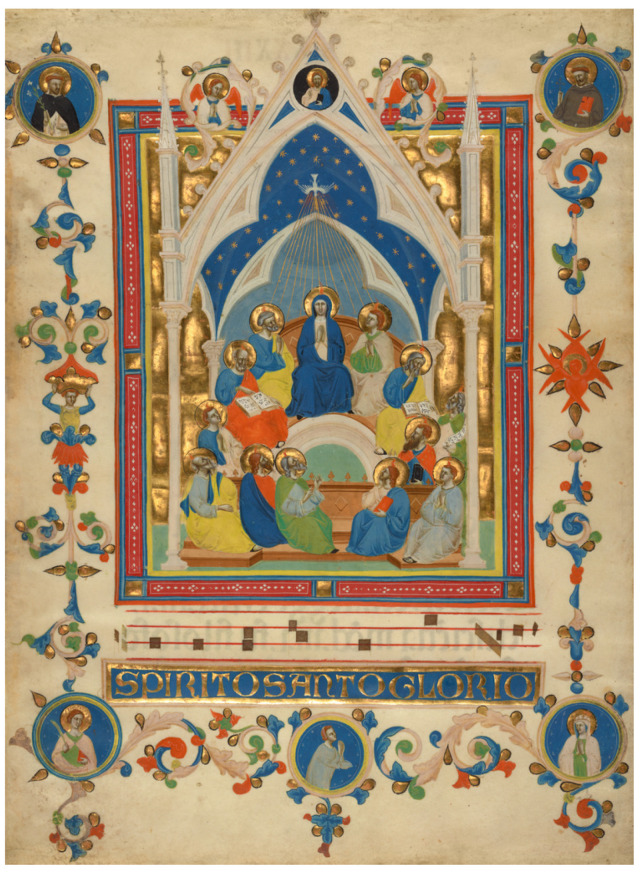
Color image of Master of the Dominican Effigies, the *Pentecost*, about 1340, The J. Paul Getty Museum, Los Angeles, Ms. 80, verso. Digital image courtesy of the Getty’s Open Content Program.

To validate the proposed system, the HSI reflectance image cube of the *Pentecost*, a 14th-Century illumination, was used. This painting has been studied in detail by conservators and conservation and imaging scientists at the National Gallery of Art, Washington DC, and the Getty Museum [10]. A complete map of the pigments present was derived by using HSI (visible-to-near-infrared (VNIR)), XRF imaging, point-fiber optics spectroscopy (350 to 2500 nm), and Raman spectroscopy. The color image of the illumination is displayed in Figure 1. The pigment database created in [10] for the development of a pigment identification neural network was used for the spectral band selection study described in Section 2.1. The database consisted of 17,000 spectra representing 25 pigments and pigment mixtures (classes) that was selected and labeled from 4 well-characterized illuminations. Collecting spectra from real works of art to create the database, instead of using spectra from mock-up samples, allows for greater spectral variability within each class.

### 2.1. Band Selection Study

Before computer processing power became adequate for fast analysis of large HSI datasets, studies to reduce the number of spectral bands for identifying materials and their conditions were common [11,12]. More recently, such studies have been used to inform band selections for MSI remote sensing applications. After testing several band selection methods, the band priority index method (BPI) [13] was chosen due to its performance on benchmark HSI datasets for remote sensing: “Indian Pines” and “Pavia University” [14]. The BPI method aims to identify the spectral bands from the HSI dataset with the largest amount of information and the least correlation. BPI applies the sequential forward search (SFS) strategy [15], which starts with an empty set of features and adds one feature per iteration to maximize a preselected “cost function.” The process is repeated until the desired number of bands is selected, where this number may be based on hardware capabilities.

**Figure 2:**
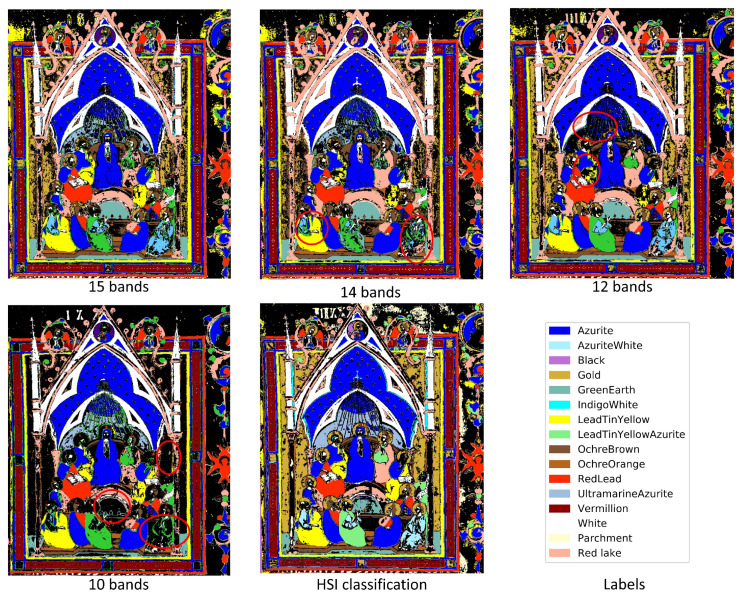
Classification results using various numbers of selected bands with BPI.

**Figure 3:**
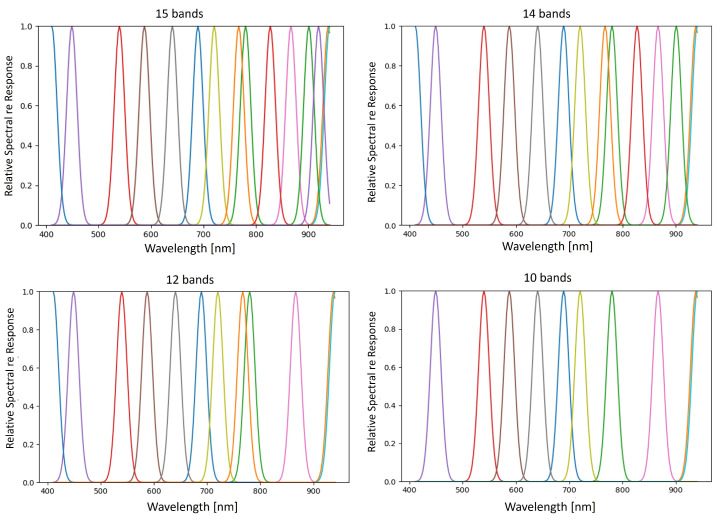
Bands chosen by the BPI method for various numbers of bands selected.

The objective function (score) of BPI is the product of the joint correlation and the variance in the band being considered for inclusion. As described by [13], the score calculated for each selected band decreases as more bands are added. If the score of a newly selected band is similar to the previous score, the new band offers little additional contribution, indicating that the number of useful bands has been determined. The BPI method was applied to the pigment database [10], and the scores suggested that 10 to 15 bands would be necessary. However, these scores do not consider the spectral profile or bandwidth of the potential MSI filters. To confirm this selection, the HSI spectra of the pigment database were resampled to simulate the proposed set of bands as Gaussian functions with an FWHM of 23.5 nm (based on available filter specifications). The one-dimensional neural network (1D-CNN) described in [10] was retrained on the spectrally reduced pigment dataset and applied to a spectrally downsampled *Pentecost* illumination for pigment classification. Classification results based on the number of bands used are shown in Figure 2, with the HSI classification result displayed on the bottom left. Due to various inconsistencies that can be seen in the 10 to 14 band classification maps (circled in red), 15 bands were chosen to use as the desired number of bands to represent the dataset. The spectral bands selected by the BPI method are displayed in Figure 3.

### 2.2. System Radiometry

The goal of the system trade study was to produce a conceptual system design for a multispectral point imaging system used in conjunction with point-XRF measurements on existing scanning easels within reasonable costs. This would enable data capture simultaneously with the XRF scans, thus reducing collection time and cost.

**Figure 4:**
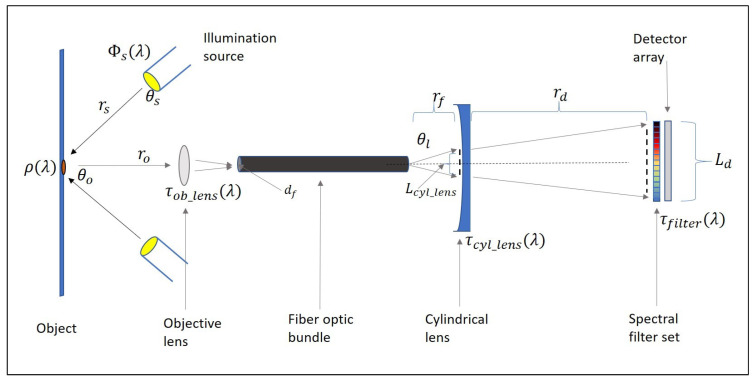
Schematic of the proposed imaging system.

**Figure 5:**
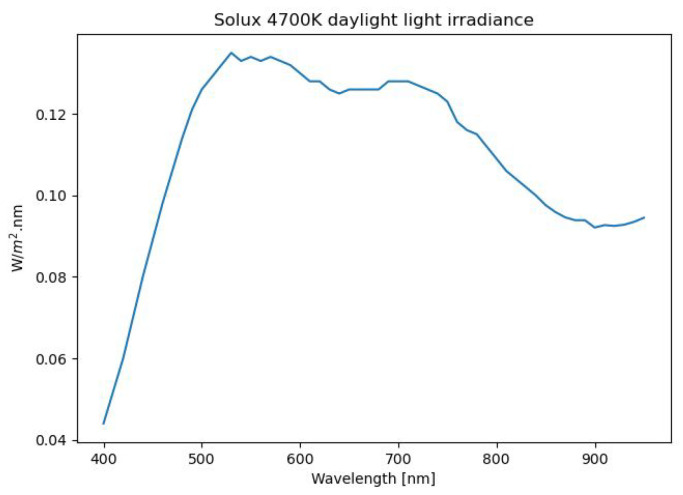
Spectral irradiance of the 50 W Solux 4700 K daylight sources.

The proposed system (see the schematic in Figure 4) consisted of 2 50-Watt (W) Solux 4700-Kelvin daylight lights. These were positioned at a distance of 1 m ($r_{s}$) from the object and angled at 45${}^{\circ }$ ($\theta_{o}$). The transmission of both the objective and cylindrical lenses are displayed respectively as $\tau_{ob\_lens}$ and $\tau_{cyl\_lens}$. Since the distance from the illumination source ($r_{s}$) to the object was more than 10-times the radius of the source, point-source illumination may be assumed. The spectral irradiance of the illumination can be seen in Figure 5 as per the supplier’s specification document.

To model the system radiometry, an average reflectance (ρ) of 0.35 was chosen, with the light reflected from a spot with a diameter of 0.125 mm. This size was determined by considering the spot size after image sampling in HSI systems currently in use in this field (as discussed in Section 2.3).

**Figure 6:**
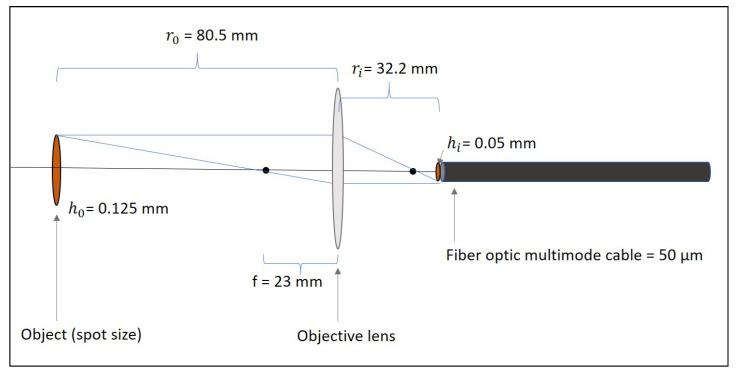
Schematic of focusing the spot onto the fiber optic cable.

The reflected light was focused into a 50 μm VIS-NIR multimode optical fiber with a 6 mm-diameter VIS-NIR coated double-convex objective lens. The distances are displayed in Figure 6.

A plano-concave cylindrical lens (focal length f=4 mm) focuses the light from the fiber onto a linear detector array that was coated with the set of bandpass interference filters specified by the band trade study. The expansion of the beam is calculated with:

(1) $$L_{d}=\left(\frac{L_{cyl\_lens}}{f}\right)\left(r_{d}+f\right)$$

where $L_{cyl\_lens}$ is calculated using the numerical aperture (NA = 0.22) of the fiber optic cable with:

(2) $$L_{cyl\_lens}=2r_{f}\left(tan\left(sin^{-1}\left(NA\right)\right)\right).$$

For the proposed system, the fiber was placed 0.25 mm from the lens, resulting in light with a diameter $L_{cyl\_lens}=0.113$ mm incident onto the lens. A linear image sensor with a length $L_{d}=3$ mm was placed at a distance of $r_{d}=102.42$ mm from the cylindrical lens. The line width at the detector was equal to $L_{cyl\_lens}=\;113$μm, which is smaller than the detector dimension of 200 μm. Thus, the system resolution was not detector-limited. Each bandpass interference filter would cover 17 detector elements of the 256 available in the array. The average transmission of these bandpass interference filters was 50%.

To estimate the spectral signals that would be captured, the radiometry of the system was modeled. The spectral radiant intensity arriving at the entrance of the detector is:

(3) $$I_{d}\left(\lambda \right)=\frac{2\cdot \Phi_{s}\left(\lambda \right)\cdot cos\theta \cdot \rho_{o}\left(\lambda \right)\cdot A_{o}\cdot \tau_{obj\_lens}\left(\lambda \right)\cdot \tau_{cyl\_lens}\left(\lambda \right)\cdot \tau_{filter}\left(\lambda \right)}{\Omega_{s}\cdot r_{s}^{2}\cdot \pi \cdot r_{o}^{2}\cdot r_{d}^{2}}\;\left(\frac{W}{sr\cdot nm}\right)$$

with the reflectance of the object $\rho_{o}\left(\lambda \right)=$ 0.35, $A_{o}$ the spot size of the image, $r_{s}$ the distance from the source to the object, $r_{o}$ the distance from the object to the lens, $\tau_{obj\_lens}\left(\lambda \right)$ and $\tau_{cyl\_lens}\left(\lambda \right)$ the spectral transmission of the objective and cylindrical lenses, and $\tau_{filter}\left(\lambda \right)$ the transmission of the filters at distance $r_{d}$.

Equation (4) is used to calculate the number of photons (digital count) arriving at the detector:

(4) $$DC=\frac{2^{bit\;depth}-1}{well\;depth}\int_{\lambda_{start}}^{\lambda_{end}}I_{d}\left(\lambda \right)\cdot \frac{QE(\lambda )}{E_{ph}\left(\lambda \right)}\Delta t\cdot dt\;\left(photons\right)$$

where QE(λ) is the quantum efficiency of the detector, $E_{ph}\left(\lambda \right)=\frac{hc}{\lambda }$ (J) is the photon energy at a specific wavelength, *h* is Planck’s constant $h=6.626\times 10^{-34}$ (J·s), *c* is the speed of light, and Δt is the integration time. The spectral limits of the integration were from $\lambda_{start}=400$ nm to $\lambda_{end}=950$ nm. The well depth specified for the detector was 50,000 electrons, and the dynamic range of the system was 12 bits (4096 gray levels)

The system noise was primarily from the variance in the signal and was modeled as a Poisson process with random variable *N* equal to the mean signal and standard deviation $\sigma =\sqrt{N}$. Additionally, the dark noise $\sigma_{dark}$, readout noise $\sigma_{read}$, and analog-to-digital conversion noise were added in quadrature to estimate the signal-to-noise ratio (SNR):

(5) $$SNR=\frac{n_{electrons}}{\sqrt{\sigma_{s}^{2}+\sigma_{read}^{2}+\sigma_{dark}+{\left(\frac{well\;depth}{\sqrt{12}\left(2^{bit\;depth}-1\right)}\right)}^{2}}}.$$

**Figure 7:**
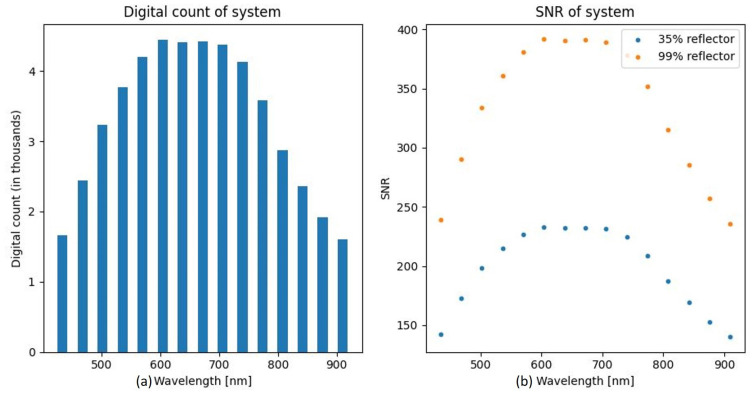
(**a**) Digital count of the modeled fixed-band system. (**b**) SNR of the modeled system.

Figure 7a displays the modeled digital count for each of the 15 bands of the system, assuming an FWHM of 23.5 nm and a 35% reflector. Figure 7b displays the spectral SNR of both a 35% and 99% reflector.

### 2.3. Image Sampling

**Figure 8:**
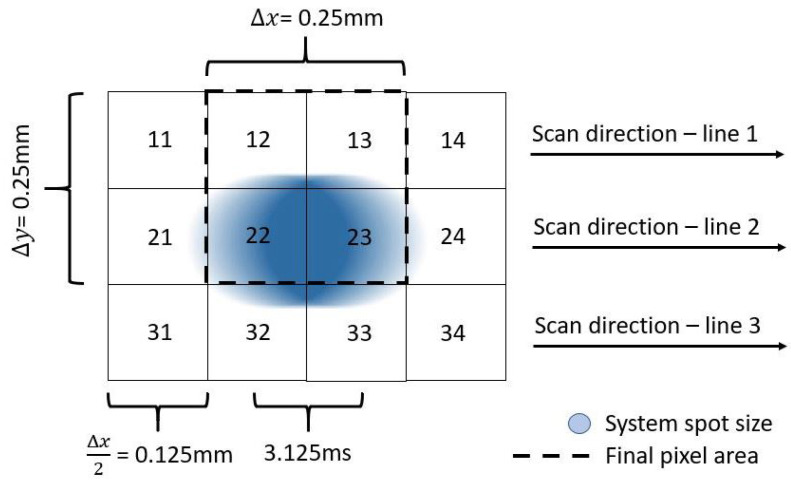
Schematic of image sampling.

The best spatial resolution currently used for HSI of paintings is of the order of 0.25 × 0.25 mm. This is the reason for the selected spot diameter of 0.125 × 0.125 mm. The collection time for each measurement would be 3.125 ms over an area of about 0.125× 0.25 mm, as per Figure 8. The blue region represents the area to be scanned during that time period, and the dashed square represents the area that was averaged to form the final 0.25 × 0.25 mm pixel. With these settings, a 1 m${}^{2}$ painting may be scanned in 28 h, which is within the acceptable range [2].

The modulation transfer function (MTF) was modeled as follows: The cylinder function that describes the beam size was convolved with a 1D rectangle function to model the motion blur. This function was convolved with the input to model the scanning, and the resulting signal was averaged over the final pixel size by convolving with a rectangle (with dimensions of the final image pixel):

(6) $$\begin{array}{c} \left(f\left[x,y\right]*\left(CYL\left(\frac{r}{\left(\frac{\Delta x}{2}\right)}\right)*\left(RECT\left[\frac{x}{\left(\frac{\Delta x}{2}\right)}\right]\cdot \left(\delta \left[y+\frac{\Delta y}{4}\right]+\delta \left[y-\frac{\Delta y}{4}\right]\right)\right)\right)\right)\\ RECT\left[\frac{x}{\Delta x},\frac{y}{\Delta y}\right]\cdot \left(\frac{1}{\Delta x}COMB\left[\frac{x}{\Delta x}\right]\cdot \frac{1}{\Delta y}COMB\left[\frac{x}{\Delta y}\right]\right) \end{array}$$

where the COMB function can be combined by substituting Δy with Δx=0.25 mm. Since the MTF differs along the two directions, the impulse response before sampling is:

(7) $$\begin{array}{c} h\left[x,y\right]=\left(CYL\left(\frac{r}{\left(\frac{\Delta x}{2}\right)}\right)\;*\;\left(RECT\left[\frac{x}{\left(\frac{\Delta x}{2}\right)}\right]\;\cdot \;\left(\delta \left[y+\frac{\Delta x}{4}\right]\;+\;\delta \left[y-\frac{\Delta x}{4}\right]\right)\right)\right)\\ RECT\left[\frac{x}{\Delta x},\frac{y}{\Delta y}\right], \end{array}$$

and the 2D transfer function is:

(8) $$\begin{array}{c} H\left[\xi ,\eta \right]=\frac{\pi }{4}\cdot {\left(\frac{\Delta x}{2}\right)}^{2}\cdot SOMB\left(\frac{\Delta x}{2}\cdot \sqrt{\xi^{2}+\eta^{2}}\right)\\ \;\;\;\cdot \left(\frac{\Delta x}{2}\cdot SINC\left[\frac{\Delta x}{2}\cdot \xi \right]\;\cdot \;2\cdot cos\left[2\pi \cdot \frac{\Delta x}{4}\cdot \eta \right]\right)\;\cdot \;{\left(\Delta x\right)}^{2}\cdot SINC\left[\Delta x\cdot \xi ,\Delta x\cdot \eta \right]. \end{array}$$

The MTF along the horizontal direction is:

(9) $$\left[\frac{H[\xi ,0]}{H[0,0]}\right]\;=\;\left|\frac{2\cdot J_{1}\left[\pi \cdot \frac{\Delta x}{2}\cdot \xi \right]}{\pi \cdot \frac{\Delta x}{2}\cdot \xi }\cdot SINC\left[\frac{\Delta x}{2}\cdot \xi \right]\cdot SINC\left[\Delta x\cdot \xi \right]\right|,$$

and along the vertical direction is:

(10) $$\left[\frac{H[0,\eta ]}{H[0,0]}\right]\;=\;\left|\frac{2\cdot J_{1}\left[\pi \cdot \frac{\xi }{\frac{2}{\Delta x}}\right]}{\pi \cdot \frac{\xi }{\frac{2}{\Delta x}}}\cdot cos\left[2\pi \frac{\eta }{\frac{4}{\Delta x}}\right]\;\cdot \;SINC\left[\frac{\eta }{\frac{1}{\Delta x}}\right]\right|.$$

**Figure 9:**
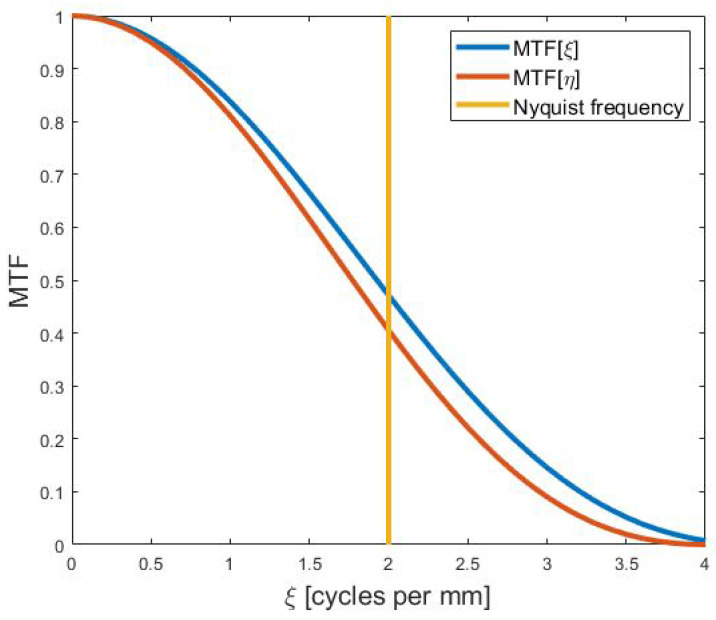
Modeled MTF of the proposed system for both directions, with the Nyquist frequency $\xi_{max}=\eta_{max}=\frac{1}{2\cdot \Delta x}=2\cdot \frac{cycles}{mm}$.

Substituting Δx=Δy=0.25 mm results in the MTF along the horizontal and vertical directions:

(11) $$\begin{array}{cc} MTF[\xi ] & =\;\left|\frac{2\cdot J_{1}\left[\pi \cdot \frac{\xi }{8}\right]}{\pi \cdot \frac{\xi }{8}}\cdot SINC\left[\frac{\xi }{8}\right]\;\cdot \;SINC\left[\frac{\xi }{4}\right]\right| \end{array}$$

(12) $$\begin{array}{cc} MTF[\eta ] & =\;\left|\frac{2\cdot J_{1}\left[\pi \cdot \frac{\eta }{8}\right]}{\pi \cdot \frac{\eta }{8}}\cdot cos\left[2\pi \cdot \frac{\eta }{16}\right]\;\cdot \;SINC\left[\frac{\eta }{4}\right]\right|, \end{array}$$

as displayed in Figure 9. The system-level MTF in the along-scan direction was only 5% lower at the Nyquist frequency than the cross-scan direction. Since the linear blur from the scan motion was half of the pixel, it was a small effect. The optical PSF was 1/4 of the pixel, so the major factor determining the system-level MTF was the pixel aperture itself. Such a high MTF at the Nyquist frequency is preferred for a system designed to collect spectra.

### 2.4. Illumination Considerations

The conservation community typically measures light exposure in units of lux, a photometric measure of luminous flux that considers the human visual response, but not IR and UV radiation, which are beyond the sensitivity of the human visual system. The analogue of illuminance in lux is:

(13) $$E_{v}\left(\lambda \right)=k\int_{400}^{950}y\left(\lambda \right)\cdot \frac{\Phi (\lambda )}{4\pi r_{s}^{2}}\cdot d\lambda$$

where y(λ) is the photopic luminosity function, Φ(λ) the spectral flux of the source, $r_{s}$ the distance from the illumination source to the object, and *k* = 683 (W/lumen) the scale factor. To include UV and IR effects, the light exposure was calculated in the same units that would have a value comparable to the suggested light level for exhibits of 50 lux per hour [16]. Thus, a week-long exhibit (40 h) would expose the object to 2000 lux. Note, using the Solux 50 W lights at a distance of one meter results in 508 lux per hour.

As previously mentioned, scanning a painting of 1 m${}^{2}$ would take approximately 28 h. Since only a very small part of the object would be illuminated at any given time, the illumination would be constrained to ensure that each area receives less than 40 min of illumination.

### 2.5. Noise-Equivalent Change in Reflectance

The noise-equivalent change in reflectance (NEΔρ) was calculated to describe the capability of the system to detect small changes in reflectance [17]. The minimum detectable change in reflectance is calculated by dividing a unit change in reflectance (Δρ) by the associated change in signal $\Delta S_{p}$ and multiplied by the noise *N* as follows:

(14) $$NE\Delta \rho =N\frac{\Delta \rho }{\Delta S_{p}}$$

The original signal was calculated using a 35% reflector, and thus, for a unit change in reflectance (Δρ=0.01), the new signal was calculated with a 36% reflector. Note that since ρ∈[0,1], Δρ=0.01.

**Figure 10:**
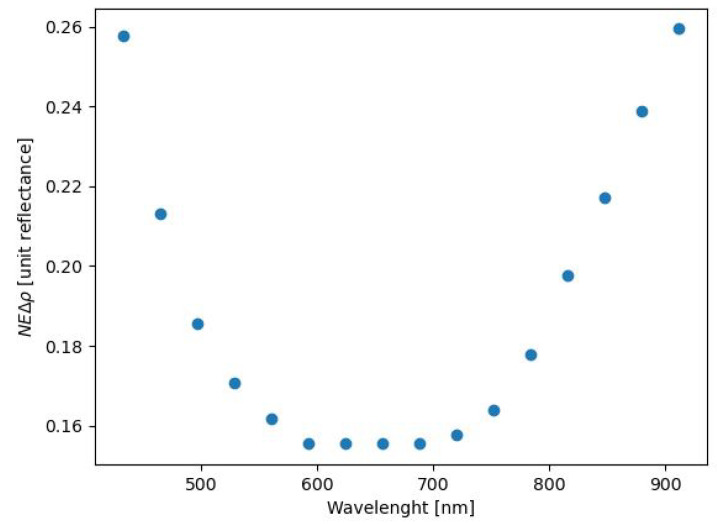
Noise-equivalent change in reflectance per unit reflectance. The signal is most easily detected in the range 600 nm <= λ <= 700 nm.

Figure 10 displays the NEΔρ for the proposed system per unit reflectance (ρ=0.01).

### 2.6. Modeling Reflectance Datacube

To visualize the results of the proposed system, the HSI reflectance values for *the Pentecost* illumination (described above) were used as the input to the radiometric model after spectral resampling of the data with the bands selected by the band selection study. A white reference target was also modeled (with reflectance set to unity) to convert the digital count to apparent reflectance. Standard calibration procedures when collecting reflectance HSI data as described by [18] were used during the data capture of the HSI datacube. However, since this was a theoretical study, we modeled perfect reflectance at 100% to convert the modeled data to reflectance.

For a more realistic output dataset, synthetic correlated noise was added to the modeled data before sampling. The synthetic noise was calculated as a two-step process as described in detail by [19]. The correlated noise was first estimated by:

- Selecting a uniform region from *the Pentecost* illumination (HSI datacube);
- Applying the minimum noise fraction (MNF) transform and retaining the noise statistics of the dataset;
- Using only bands with eigenvalues greater than 2.0 for the inverse transform of the data back to the original data space (so that the result was essentially noise-free);
- Subtracting the noise-free data from the original selected uniform reflectance data (to produce an estimated dark scan).

Then, after creating the estimated dark scan, the synthetic correlated noise cube was created:

- PCA was applied to the estimated dark scan to decorrelated the noise;
- The standard deviation of each transformed band was measured;
- Gaussian noise images with zero mean and the extracted standard deviation were generated for each band (scaled according to the output image size required);
- The inverse PCA transform was evaluated for the generated noise images using the same statistics present in the forward transform.

The resulting correlated noise cube was sampled to obtain the MSI bands, which were added to the modeled reflectance image cube.

**Figure 11:**
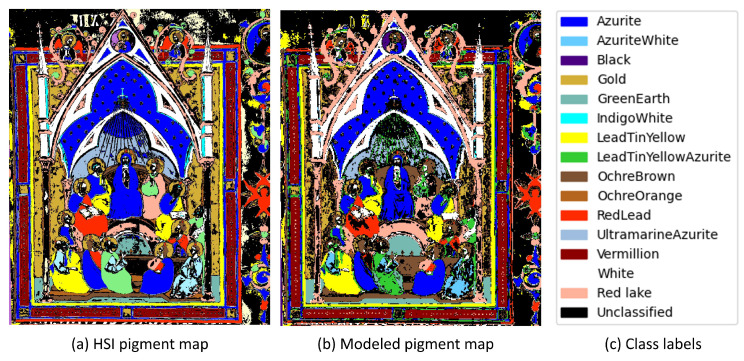
Pigment maps comparing the (**a**) modeled classification map to the (**b**) classification map created using the HSI data. The associated pigment labels are displayed in (**c**).

To test the output of the modeled datacube, the 1D-CNN created for testing the 15-band BPI method was applied to the noise-added, modeled reflectance cubes. The classification results can be seen in Figure 11.

**Figure 12:**
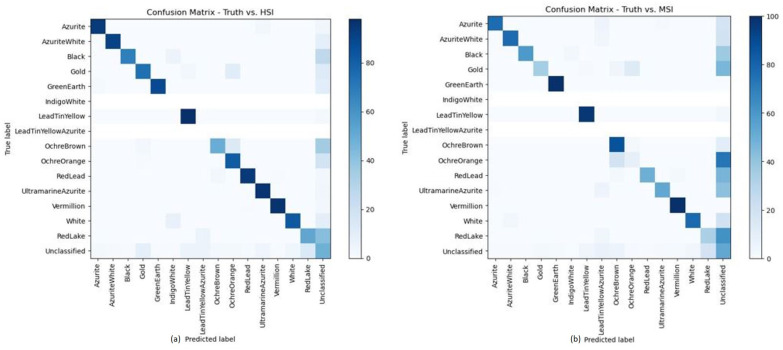
Confusion matrices comparing the (**a**) HSI pigment classification and (**b**) the modeled classification map to the ground truth pigment data.

For a more quantitative analysis, confusion matrices of the HSI pigment map and the modeled pigment map as compared to the ground truth data are displayed in Figure 12. Confusion matrices are a well-known metric for visualizing the performance of machine learning models [20]. Note, all pigments and mixtures found in the ground truth map that were not part of the original training database were excluded from the confusion matrix. Additionally, due to unbalanced class sizes, each pigment is displayed as a percentage of the ground truth value. This ensured that the data of all classes are clearly displayed and not just that of the larger classes.

## 3. Results

The HSI reflectance values of the *Pentecost* illumination were used to test the proposed system, as described in the previous section. This illumination was chosen for modeling due to the extensive ground truth data available to validate the findings. This does not represent the actual spatial resolution of the modeled system because the datacube was sampled to mimic image capture and motion blur, as per Section 2.3.

**Figure 13:**
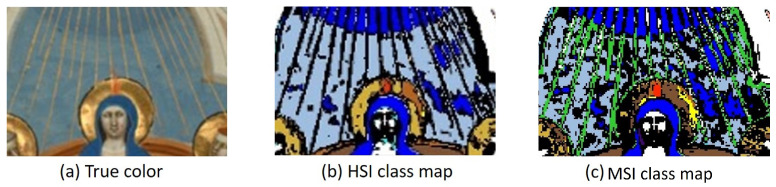
Detail of the *Pentecost* (**a**) and associated classification maps created using the HSI data (**b**) and the MSI data (**c**).

Figure 11 displays the classification results obtained using (a) the full HSI reflectance data and (b) the modeled MSI reflectance data. The associated pigment labels are listed on the right. Since conservation scientists are not interested in a pixel-by-pixel ground truth, but instead, in general pigment maps that specify the locations of the pigments or mixtures, the results were compared visually and not statistically. In general, the modeled MSI classification map displays only minor differences to the HSI classification map. For example, the allocation of lead tin yellow/azurite lines (between the ultramarine/azurite background) should have been allocated as gold. This can be seen in the detail in Figure 13.

In the field of art conservation science, quantitative analysis is not often used as conservators do not work on a pixel-by-pixel basis. Thus, classification maps as displayed in Figure 11 are of much greater value for the purpose of conservation. However, for completeness, the confusion matrices of Figure 12 are displayed to show the effectiveness of the proposed system. As mentioned, not all classes are shown here, as the neural network could not predict classes that were not part of the original training set. Looking at the matrices, one can see minor differences between the two comparisons.

**Figure 14:**
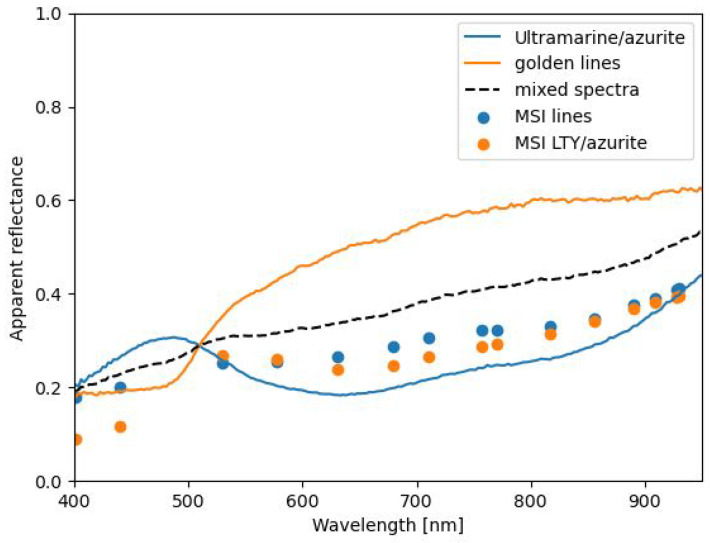
Spectra taken from the HSI and MSI *Pentecost* datacubes. The black dashed line displays the binned spectra when ultramarine/azurite and gold spectra are adjacent. The blue dots represent the MSI spectra of the lines, and the orange dots represents the matched MSI spectra of lead tin yellow/azurite.

Looking at the spectra of the associated pigments in Figure 14, the differences could potentially be explained. Due to the pixel sampling method used to create the MSI images, the thin lines captured by the HSI system are mixed spatially with the ultramarine/azurite background. The solid orange line in the graph represents the HSI spectra of the golden lines (see the detail of Figure 13a). The solid blue line is the ultramarine/azurite HSI signal, and the dashed black spectrum represents a mixture of the ultramarine/azurite and golden line spectra. The blue dots depict the MSI spectra at those lines, while the orange dots display an MSI spectrum taken from the correctly allocated lead tin yellow/azurite green robe.

This incorrect allocation is understandable when comparing the similarity of the MSI spectra. However, this specific misallocation should not be a limitation for actual data collection, as the spot size of the HSI reflectance datacube used in this analysis was slightly larger than 0.3 × 0.3 mm, resulting in a modeled MSI image of a 0.6 × 0.6 mm resolution. This is much larger than the proposed system resolution.

**Figure 15:**
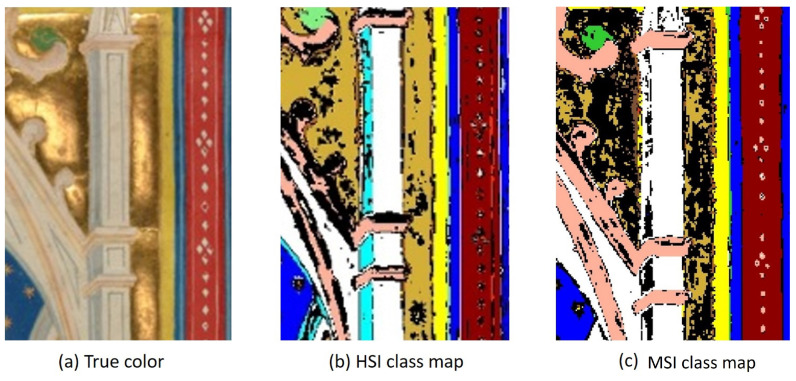
Detail of the *Pentecost* (**a**) and associated classification maps created using the HSI data (**b**) and the proposed system (**c**).

Another noticeable difference between the HSI and modeled MSI class maps is the white spire in Figure 15. However, the HSI classification of indigo (the “light blue” in the spire) is incorrect based on the ground truth data collected, which also indicates the presence of some copper-containing blue. The MSI classification of white coincides with the ground truth map [10].

## 4. Discussion

The aim of this study was to design a low-cost solution for museums that already have motorized XRF scanning easels such that the proposed multispectral imaging system can be attached for simultaneous image capture. Since macroscale XRF imaging already takes a significant amount of time, collecting reflectance data concurrently with XRF frees up time for the conservation scientists, as well as limits the time the object is kept in the lab. Consequently, with the proposed system, for minimum cost and effort, a second imaging modality can be used to collect useful multispectral data in addition to the XRF collection. The results showed that for appropriately selected spectral bands, the images captured by the proposed system can produce pigment maps and classification results comparable to those obtained using data from a hyperspectral imaging system.

The modeled system produced data with an SNR of just under 400 for a 99% reflector for spectral bands between 600 and 700 nm. This is comparable to the results obtained with high-end HSI systems currently used in this field, which typically specify an SNR of 400 for a 99% diffuse reflector [21]. Looking at the predicted NEΔρ plot in Figure 10, a reflectance change of 0.0026 would just be detectable above the noise for the 432 nm wavelength. This smallest possible reflectance change decreased significantly for wavelengths between 600 and 700 nm. This parameter indicates that the proposed system is very sensitive to minor changes in reflectance and as such would be useful in detecting potentially very subtle changes in reflectance due to the pigment material properties.

A limitation to this design was the pre-defined filters that were chosen based on a specific pigment dataset. Paintings and illuminations from other time periods, locations, or workshops would potentially have different pigments, mixtures, and/or binders. Thus, different sets of MSI bands would have to be selected for each new pigment dataset, and the filters changed accordingly. Another option would be to increase the number of filtered detectors in the system (to be sensitive to a wider range of pigment properties) and then select which bands to analyze based on knowledge of the pigments in the work. A third potential alternative would be to use a linear variable bandpass filter, which produces a continuous spectrum across the spectral response of the filter, similar to a hyperspectral sensor, where bands can be selected after image capture. A current shortcoming of this however is that the currently available linear variable filters do not span the full 400 to 950 nm spectral range. Any of the above-mentioned options for detecting the spectral signature would still leverage the point-scanning and optical system of the currently proposed system, producing a spectral image for use in pigment mapping of works of art concurrently with the XRF scanning.

## Data Availability

Not applicable.
